# First molecular and isotopic evidence of millet processing in prehistoric pottery vessels

**DOI:** 10.1038/srep38767

**Published:** 2016-12-22

**Authors:** Carl Heron, Shinya Shoda, Adrià Breu Barcons, Janusz Czebreszuk, Yvette Eley, Marise Gorton, Wiebke Kirleis, Jutta Kneisel, Alexandre Lucquin, Johannes Müller, Yastami Nishida, Joon-ho Son, Oliver E. Craig

**Affiliations:** 1 Department of Archaeological Sciences, University of Bradford, Richmond Road, Bradford BD7 1DP, UK; 2 BioArCh, University of York, Heslington, York YO10 5DD, UK; 3 Instytut Prahistorii, Adam Mickiewicz University in Poznań, Wieniawskiego 1, 61-712 Poznań, Poland; 4 Center for Integrative Geosciences, University of Connecticut, Storrs, Connecticut, 06269, USA; 5 Institute of Pre- and Protohistoric Archaeology, Johanna-Mestorf-Straße 2-6, Christian-Albrechts-Universität zu Kiel, Kiel, Germany; 6 Niigata Prefectural Museum of History, Sekihara 1, Nagaoka, Niigata 940-2035, Japan; 7 Department of Archaeology and Art History, Korea University, 2511 Sechong-ro, Jochiweon-up, Sejong-si, 339-700, South Korea

## Abstract

Analysis of organic residues in pottery vessels has been successful in detecting a range of animal and plant products as indicators of food preparation and consumption in the past. However, the identification of plant remains, especially grain crops in pottery, has proved elusive. Extending the spectrum is highly desirable, not only to strengthen our understanding of the dispersal of crops from centres of domestication but also to determine modes of food processing, artefact function and the culinary significance of the crop. Here, we propose a new approach to identify millet in pottery vessels, a crop that spread throughout much of Eurasia during prehistory following its domestication, most likely in northern China. We report the successful identification of miliacin (olean-18-en-3β-ol methyl ether), a pentacyclic triterpene methyl ether that is enriched in grains of common/broomcorn millet (*Panicum miliaceum*), in Bronze Age pottery vessels from the Korean Peninsula and northern Europe. The presence of millet is supported by enriched carbon stable isotope values of bulk charred organic matter sampled from pottery vessel surfaces and extracted *n*-alkanoic acids, consistent with a C_4_ plant origin. These data represent the first identification of millet in archaeological ceramic vessels, providing a means to track the introduction, spread and consumption of this important crop.

Millet, a small-grained cereal with glumes of hard silica-rich tissue and a short growing season, was an important prehistoric crop that became established across Eurasia. However, the origins and dispersal of domesticated millet and the motivations for its incorporation into cropping regimes are far from clear. The most important species of millet cultivated in Eurasia are common or broomcorn millet (*Panicum miliaceum*) and foxtail millet (S*etaria italica*). By 6000 BC, based on charred grain assemblages, millets appear to have been cultivated across several northern Chinese cultures stretching some 1500 km along the Yellow River from the Xinglongwa Culture of the northeast in Manchuria, the Cishan Culture (Hebei Province), the Peiligang Cultures in northern Henan, the Houli Culture in northwest Shandong and in southeast Gangsu with the Dadiwan Culture^1^,^2^,^3^,^4^,^5^. Earlier dates for domestication at Cishan, based on starch grain and phytolith morphology^6^,^7^, have been questioned^3^,^4^. Where human remains are available, stable carbon isotope analysis of bone collagen has proved important in establishing the contribution of millet, a C_4_ plant, to diet in the period after domestication. The current isotope data show a mixed picture with millet appearing to range in the diet from a minor food component to a major food resource^8^.

From the presumed centre of origin in northern China, both millets are subsequently found over large areas of Eurasia, although the earliest finds are mostly of broomcorn millet^4^. Evidence from charred millet grain and stable isotope analysis of bone collagen from human and faunal remains has shown that broomcorn millet was established at Begash in Kazakhstan by the late third millennium BC^9^,^10^, in Eastern and Central Europe by ca. 1,500 cal BC^11^,^12^,^13^,^14^ and in northern Central Europe not before 1,200 cal BC^15^,^16^,^17^,^18^,^19^. Direct ^14^C dating of millet grains in Europe, considered to be found in much earlier contexts, has shown that they date to the second millennium BC or later^20^. In Europe, millet cultivation developed much later than the earliest agriculture at the onset of the Neolithic prompting debates that seek to answer the question ‘Why move starch into areas where starch was already available?’^12^,^21^,^22^.

The dispersal of millet may have been facilitated by mobile hunters, foragers and pastoralists occupying temporary or seasonal settlements since there are clear practical and logistical benefits due to its short growing season and ability to grow in marginal soils with low rainfall. In agrarian societies, millet may have been integrated into flexible crop regimes enabling an extended growing season and a consequent increase in food security^8^,^19^,^21^. In contrast Boivin and co-workers^22^ emphasize the social factors involved in the adoption of millet cultivation, noting that the perceived value of millet as an exotic crop may have been important in early exchange systems, well before the crop became established as a staple food. However, missing from this debate is any knowledge of how millet was prepared and consumed. This is essential to understand its perceived value and evolving role as a foodstuff.

Evidence from material culture has rarely been deployed in discussions involving the presence and role of specific crops, such as millets, in early societies. Impressions on pottery vessels, thought to be of millet grains, have occasionally been reported^23^. Molecular and isotopic analysis of organic residues associated with pottery containers has developed as a powerful tool for the direct identification of vessel use and insight into diverse cultural practices^24^. A range of plant products, including oils, resins and waxes has been recognised. Detecting residues of grain or seed crops in pottery vessels has proved more challenging. One exception is the presence of the C4 crop maize in vessels from North America utilising a combined lipid biomarker and compound-specific carbon isotope approach^25^,^26^. Other approaches have been explored in order to document the occurrence of millets in the archaeological record. The most promising is molecular analysis of soils and sediments demonstrating associations with millet cultivation in pit fills or sediment cores^7^,^27^,^28^,^29^; although this approach provides little evidence as to how the crop was processed or consumed.

Here, we propose a new approach for studying millet utilisation in the past, through its identification in direct association with archaeological pottery vessels using a combination of bulk isotope analysis, compound-specific carbon isotope analysis and identification of the pentacyclic triterpene methyl ether (PTME), miliacin. Miliacin is the principal (c. 99%) PTME in broomcorn millet (*P. miliaceum*) but is absent in many other commonly cultivated species or present in similar abundance to other PTMEs^28^. It is abundant in other *Panicum* species but occurs with high abundances of amyrin methyl ethers. Miliacin is absent in *Setaria italica* (foxtail millet) and is not ubiquitous in Pennisetum. The strength of this approach is that only a small number of the C4-grasses of the Panicoideae subfamily synthesize miliacin as their sole PTME^28^. The presence of high abundances of miliacin in sediment profiles indicating the locations of past millet cultivation has already been demonstrated^27^. In this study of Alpine lake sediments, *P. miliaceum* was deemed to be the only possible source of miliacin, given the context and since it was the only PTME detected. High abundances of miliacin have also been reported in Early Iron Age palaesols (eastern Ukraine) from pits comprising broomcorn millet crop processing waste^29^. In contrast, a study of pit fills from Neolithic Cishan, China identified miliacin in authentic samples of both *P. miliaceum* and *S. italica* and suggested differences between the two species based on the presence of other PTMEs in lower abundance^7^. The finding of miliacin in *S. italica* is not supported by investigations of authentic samples^28^.

Unlike previous studies we focus on the identification of millet in pottery using molecular and isotopic characterisation. Ceramic vessels are abundant on archaeological sites during the period of millet domestication and dispersal. Prehistoric Eurasian pottery has been studied by archaeologists for centuries and often benefits from well-established chronologies. This approach also provides a means of contextualizing millet within the spheres of culinary practice and artefact use, allowing further and more detailed insight into the cultural significance of the crop.

## Archaeological samples

**Figure 1:**
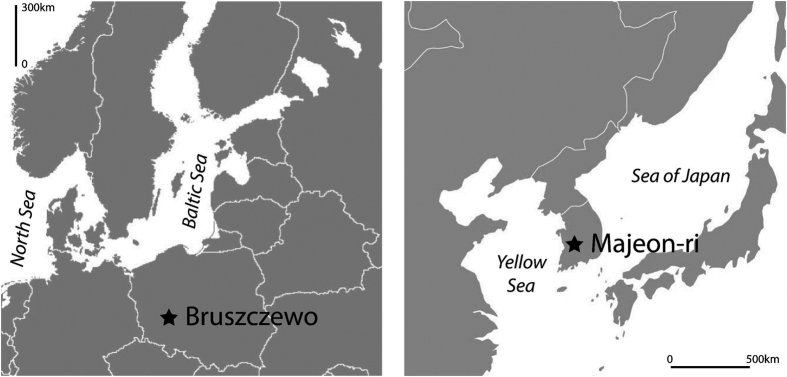
Location of sites investigated in Europe and East Asia (Adobe Illustrator CS4 14.0.0. http://www.adobe.com/uk/).

Ceramics from two prehistoric sites, one in East Asia and one in Europe, were selected for analysis in order to demonstrate the approach (Fig. 1). These sites were chosen as charred millet grains were identified amongst the botanical remains at both sites.

Bruszczewo, a settlement in Poland some 60 km south of Poznań (52°00′47″N, 16°35′07″E), has been subject to comprehensive investigations^30^,^31^,^32^,^33^. Situated on a spur and close to a lake, two main settlement periods each with several sub-phases can be distinguished: An Early Bronze Age phase (EBA, 2100–1650 cal BC) with fortification, houses and clear settlement activities, and after an apparent gap of several hundred years, occupation dated to the Late Bronze Age/Early Iron Age (LBA/EIA, 1100–800 cal BC). The excavations uncovered several EBA layers and houses preserved in a wet area below the spur at the shore of the lake that date to ca. 1800–1650 BC cal^33^. Constructed originally on dry ground at the lakeshore during the EBA, the water level rose in the Middle Bronze Age and preserved parts of the site under wet conditions^34^. Archaeobotanical analyses revealed a diachronic differentiation in the occurrence of broomcorn millet. Only single charred grains were recovered from the EBA contexts, but they are numerous in the LBA thus indicating its relevance as food crop in this phase. Furthermore, there are impressions of millet grain in some sherds, particularly on the interior surface of the lower portion of the vessel, perhaps to facilitate the drying process prior to firing. Sixty-one visible charred deposits or ‘foodcrusts’ adhering to pottery vessel surfaces were sampled. The samples include those from clearly defined and dated EBA and LBA/EIA contexts as well as a mixed transgression layer from the lake. This layer includes significant EBA material and very few younger finds; hence this group is identified as ‘mostly EBA’.

The Majeon-ri site (36°09′89″N, 127°08′37″E) is a settlement with a cemetery and paddy field system with wooden structures belonging to the Late Bronze Age and dated to 800–500 cal BC, located in the western region of the Korean peninsula^35^. Fifteen pottery sherds excavated from the irrigation ditch and puddle features at this site were selected for analysis. Unlike the pottery sherds from Bruszczewo, no charred surface deposits were observed.

## Results

### Mobilisation and absorption of miliacin in pottery

To test whether miliacin is mobilised from millet during processing and absorbed into the ceramic matrix, an experiment was undertaken involving the prolonged cooking of four species of millet; broomcorn millet (*Panicum miliaceum*), finger millet (*Eleusine coracana*), barnyard millet (*Echinochloa esculenta*) and foxtail millet (*Setaria italica*) in replica ceramic vessels. The results performed on solvent extracts of powdered sherds from these vessels show that a low quantity of miliacin (290 ng g^**−1**^ ceramic) is transferred to the pottery vessel wall during cooking of *P. miliaceum*. No miliacin was detected in the three other millet varieties, confirming previous analysis made directly on the plants^28^. This study reports yields of 297–476 μg miliacin g^−1^ *P. miliaceum* grain following solvent extraction and column chromatography to isolate the neutral fraction^28^. Much lower levels of miliacin were found in the leaves, roots and stems of *P. miliaceum* and the compound was found to be absent in the hulls. It is therefore likely that only a small proportion of miliacin is released and transferred to the ceramic matrix during cooking of millet grain.

### Bulk carbon and nitrogen isotope analysis of Bruszczewo charred surface deposits

**Figure 2:**
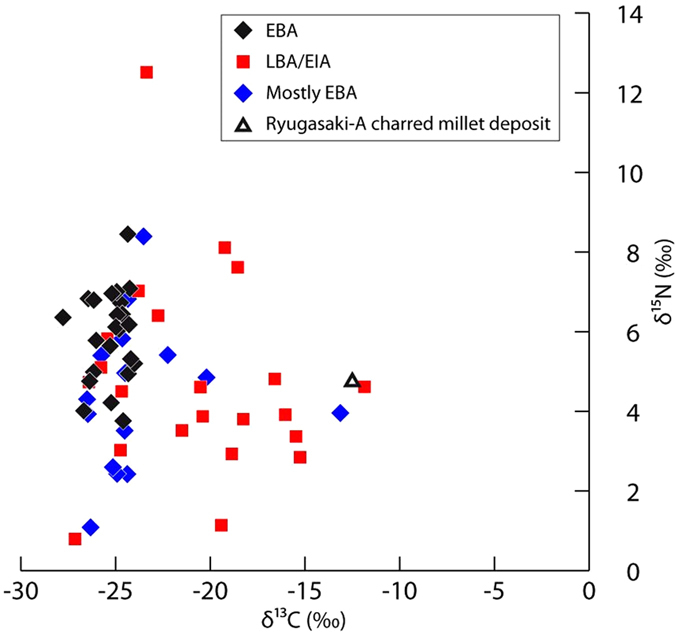
Plot of δ^15^N versus δ^13^C values for foodcrusts adhering to the interior walls of pottery vessels from Bruszczewo. The result from a single foodcrust containing carbonized millet grain from Japan (Ryugasaki) is also plotted for comparison^37^.

The presence of charred surface deposits at Bruszczewo permitted the application of elemental analysis isotope ratio mass spectrometry (EA-IRMS) to determine bulk carbon (δ^13^C) and nitrogen (δ^15^N) isotope ratios. Figure 2 shows a marked change in carbon isotope ratios between the EBA (and ‘mostly EBA’) and LBA/EIA phases with indications in the latter of a wider range of foods including a possible C_4_ plant input (see also Table 1 and Supplementary Table 1). The EBA samples form a clustered group considered to represent C_3_ plants and animals consuming C_3_ plants, relatively depleted in ^13^C. In contrast the LBA/EIA samples plot over a much wider range of carbon isotope values with 45% of the samples having δ^13^C values greater than −20.0‰, consistent with the input of ^13^C-enriched C_4_ plants such as millet. Any input from marine foods is highly unlikely as a result of the distance from the coast, absence of marine fish bone in the faunal assemblage, the low δ^15^N values and the high C/N ratios. The difference in carbon isotope ratios between the EBA and LBA/EIA samples is significant (heteroscedastic two-tailed t-test, p = <0.05). The δ^15^N values are not significantly different. The low δ^15^N values and high C:N values in the majority of samples suggest the presence of low trophic level foods expected from the charring of plant tissues in the pots^36^.

In general, the LBA/EIA foodcrusts from Bruszczewo have lower δ^13^C values when compared with direct measurements on charred millet grains suggesting that mixtures of C_3_ and C_4_ resources were processed in some of the vessels. For example, bulk isotope data (δ^13^C = −12.5‰; δ^15^N = 4.7‰, Fig. 2) was obtained on a deposit comprising carbonized millet grains adhering to a potsherd from Ryugasaki A (Shiga Prefecture, Japan) and radiocarbon dated to 800–555 cal BC^37^. Bulk carbon isotope analysis of charred broomcorn and foxtail millet grain from archaeological sites in northern China gave mean values of −11.1 ± 0.5‰ (*n* = 26) and −10.5 ± 0.6‰ (*n* = 27) respectively^38^. Direct ^14^C dates (*ca*. 1500 BC to 500 AD) determined on charred *P. miliaceum* grains from central and southeastern Europe yielded δ^13^C values from −9.6 to −11.2‰^20^. Data from cooking experiments in pottery vessels show relatively minor (*ca*. 1‰) shifts in bulk isotope values of foods during cooking, including millet grain^39^ and this is consistent with heating experiments undertaken directly on cereal grains^40^. These data are helpful when evaluating bulk isotope data of ‘foodcrusts’ transformed by heating and suggest that changes due to cooking are unlikely to impact on the differences in carbon isotope values between C_3_ and C_4_ plants. Assessing the proportion of C_3_ and C_4_ (maize) foods incorporated into charred deposits has been attempted but cooking experiments and modelling of the data highlights caution in interpreting bulk carbon isotope data in this way^41^,^42^.

### Molecular characterisation by gas chromatography-mass spectrometry (GC-MS)

Two of the ‘foodcrusts’ from Bruszczewo, one EBA and one LBA/EIA, were selected for GC-MS and GC-c-IRMS (Table 2). They were chosen to represent widely separated carbon isotope values (−26.0‰ for the EBA sample and −11.8‰ for the LBA/EIA sample). The GC-MS data are summarised in Table 3. Despite markedly different bulk carbon isotope ratios, the lipid composition of the samples is very similar. Both residues are characterized by a narrow range of *n*-alkanoic acids (C_14:0_ - C_18:0_) with a high C_16:0_ over C_18:0_ abundance. The presence of C_16_ and C_18_ ω-(o-alkylphenyl)alkanoic acids was detected in both samples. The C_18_ analogues are clearly visible in the total ion current (TIC) chromatogram of the LBA/EIA sample whereas in the EBA sample they were only detected in the extracted ion chromatograms (*m/z* 105 and 290). These ω-(o-alkylphenyl)alkanoic acids are thermal alteration products of unsaturated *n*-alkanoic acids^43^. The distribution of the C_18_ acids is consistent with those of authentic heated plant oils and not consistent with the profile given by a terrestrial animal fat and aquatic oil^44^. No trace of C_20_ and C_22_ analogues, commonly associated with the thermal alteration of longer-chain *n*-alkenoic acids in aquatic oils^41^, was detected. In summary, the lipid extracts are consistent with a plant origin but further differentiation is not possible. Epicuticular waxes, phytosterols and aquatic biomarkers are absent. Miliacin was detected by GC-MS operating in SIM mode in the LBA/EIA sample, suggesting the presence of broomcorn millet, but absent in the EBA sample.

**Figure 3:**
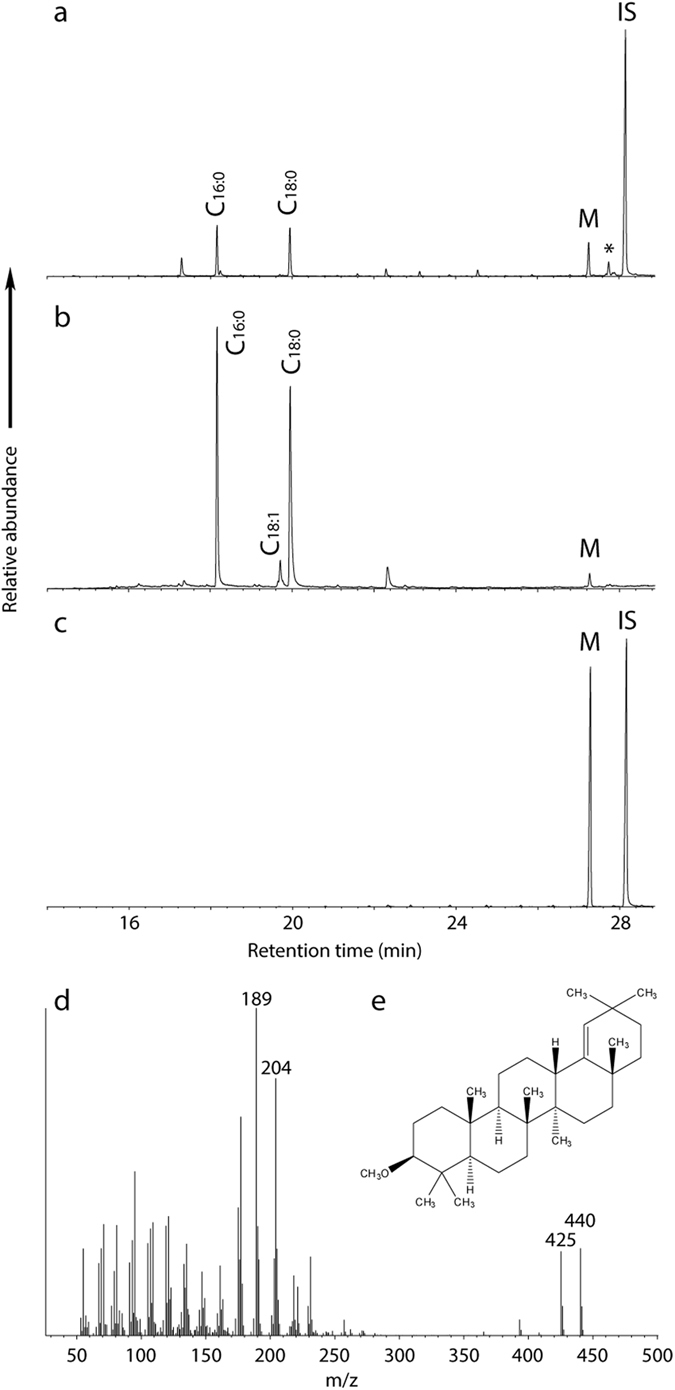
Identification of miliacin (olean-18-en-3β-ol ME) by GC-MS, (**a**) Partial total ion current (TIC) chromatogram of the archaeological sample from MJR10 (Majeon-ri). (**b**) Partial TIC chromatogram of the modern broomcorn millet cooking experimental sample. (**c**) Partial TIC chromatogram of the authentic miliacin. (**d**) Mass spectrum of miliacin. (**e**) Chemical structure of miliacin. Peak identities - M: miliacin, *: stigmastanol (trimethylsilyl ether) IS: internal standard (*n*-hexatriacontane).

Lipids were detected in all of the samples from Majeon-ri (*n* = 15). Separate solvent and acid/methanol extracts are dominated by saturated *n*-alkanoic acids (Supplementary Table S2). A range of other compounds were detected in the residues including *n*-alkanols, ketones and sterols/stanols. The presence of levoglucosan in MJR03 supports the cooking of plant tissues since this molecule is formed from the pyrolysis of starch and cellulose^45^. In addition, C_18_ ω-(o-alkylphenyl)alkanoic acids were detected in two samples, MJR06 and 08. Miliacin was identified in seven of the 15 sherds from Majeon-ri. In five of these cases, the molecule was identified in both acidified methanol and solvent extracts obtained on samples taken from the same sherd. In the remaining two sherds, miliacin was only identified in the acidified methanol extract, presumably due to the higher recovery of lipid using this extraction methodology. Figure 3a shows the presence of miliacin in extracts from sample (MJR10). The mass spectrum (Fig. 3d) is indicative of a pentacyclic triterpene methyl ether (M^+^ *m/z* 440; base peak *m/z* 189). The retention time of this peak matched an authentic sample of miliacin (Fig. 3c) as well as the peak corresponding to miliacin released experimentally by boiling broomcorn millet (Fig. 3b).

### Compound-specific carbon isotope analysis of n-alkanoic acids by GC-combustion-isotope ratio mass spectrometry (GC-c-IRMS)

The most frequent compounds usually encountered in archaeological cooking vessels are *n*-alkanoic acids (fatty acids). However, C_3_ and C_4_ plants cannot be distinguished solely on their *n*-alkanoic acid distributions^25^. In view of this, the compound-specific carbon isotope compositions of the most abundant *n*-alkanoic acids in the lipid extracts were determined by GC-c-IRMS. Table 3 presents the data obtained on the two samples from Bruszczewo compared with the bulk carbon isotope data. As expected, the lipids are more depleted in ^13^C than the bulk samples, which may contain degraded proteins and carbohydrates relatively enriched in ^13^C. However, there is a marked difference in the offset between the bulk and compound-specific carbon isotope values (Δ_BULK-CSIA_) of the two samples. The magnitude of the offsets fall within the range determined from modern bulk and compound-specific carbon isotope data from C_3_ plants (i.e., 3–9‰) and C_4_ plants (i.e., 7–14‰) respectively^46^. These data, together with the lipid molecular information, suggest that the residues are dominated by a single foodstuff – a C_3_ and a C_4_ plant (millet) respectively.

Compound-specific carbon isotope analysis was also undertaken on seven lipid extracts from Majeon-ri where sufficient quantities of *n*-alkanoic acids were preserved (Supplementary Table 2). Miliacin had been identified by GC-MS in four of these samples. Three samples (MJR06, MJR09 and MJR10) contained *n*-alkanoic acids relatively enriched in ^13^C in keeping with the processing of C_4_ plants such as broomcorn and foxtail millet. However, miliacin was identified in only two of these, perhaps reflecting differential preservation or that another species of millet, such as foxtail millet, was predominantly processed in this vessel. Conversely, despite the presence of miliacin, two of the samples (MJR02, MJR08) have more mixed fatty acid δ^13^C values indicating a combination of C_3_ and C_4_ resources.

## Discussion

The presence of miliacin as the only PTME in the ceramic lipid extracts strongly suggests the presence of broomcorn millet processed in pottery vessels from the Korean and European Bronze Age. This represents the first molecular identification of this plant in archaeological pottery vessels opening up the potential for further research. At Bruszczewo, the high δ^13^C and low δ^15^N values of the visible surface deposits and the larger offset between the bulk and compound-specific carbon isotope values are consistent with C_4_ plant signals. The presence of charred deposits and thermally altered lipids also implies that millet was heated in the preparation of the foodstuff for consumption. The presence of millet in the LBA/EIA samples is supported by archaeobotanical evidence from the site. A few charred millet grains have been recovered from EBA layers, which could point to small-scale cultivation whereas abundant charred millet grains have been recovered in LBA/EIA contexts. However, caution is required given the possibility of movement of small grains through the soil column^20^. The archaeobotanical evidence suggests that millet became a widespread and important crop during the LBA/EIA of Eastern, South-eastern, Central and northern Central Europe^14^,^15^,^16^.

At Majeon-ri, the occurrence of miliacin in the majority of the sherds analysed (Supplementary Table 2) indicates that broomcorn millet was regularly processed in these vessels. It has been suggested that human diet in the Korean Bronze Age was largely based on C_4_ plants. For example, carbon isotopic analysis of human bone collagen from the contemporary Konam-ri site on the west coast of Korea produced values consistent with C_4_ plant consumption^47^. However, consumption of marine resources, also enriched in ^13^C, complicates this interpretation. Whilst the identification of millet in pottery does not allow dietary quantification, it does demonstrate that millet was directly consumed in Bronze Age Korea. Further insights into how millet was consumed are also provided by the analysis. For example, variability in δ^13^C values of alkanoic acids extracted from the vessels containing miliacin shows that millet was commonly mixed, or sequentially processed, with other food products. This may indicate it was not treated specially or, at least, afforded dedicated ceramic vessels for its preparation. The presence of thermally-altered plant derived compounds in the potsherds, such as C_18_ ω-(*o*-alkylphenyl)alkanoic acids and levoglucosan shows that millet was heated during its preparation. Millet is often used as gruels and porridge or ground into flour by contemporary societies^11^. Another potential role for millet is as a base for fermented beverages. For example, *Boza* is a slightly alcoholic (1.5%), sour drink made without any additional malt prepared by grinding the millet grains, mixing with water, cooking and fermentation^48^. Distinguishing a gruel or porridge from a cooked and fermented millet-based drink is not possible from the data presented here but further experimental work, consideration of vessel typologies and analysis of vessels from ethnographic contexts may help elucidate these practices in the past.

The identification of millet associated with pottery provides a new opportunity to examine the history of this important crop. Firstly, this approach has the advantage of identifying direct consumption of millet, a distinction that is not afforded by most archaeobotanical finds and particularly pertinent since millet is often considered to be an important fodder crop^11^. Secondly, the identification of millet in pottery provides unique information regarding its mode of preparation and consumption, which may be crucial for understanding the role and value of the crop when first introduced to a region^21^,^22^. Given that the wild ancestor of broomcorn millet has not yet been identified, pottery residue analysis could help to identify the first intensive use of this plant. Thirdly, the abundance of pottery already recovered from archaeological sites, and held in collections, provides an easily accessible resource for undertaking a wider survey of the introduction and spread of millet cultivation and the role it played in food processing and diet across Eurasia.

## Methods

### Production of experimental vessels

Experimental vessels (h. 30 cm, d. 28 cm, 7 mm thickness) were formed by coiling, and fired at a temperature of 1,000 °C using an electric kiln. These vessels were used to cook four varieties of millet; broomcorn millet (*Panicum miliaceum*), finger millet (*Eleusine coracana*), barnyard millet (*Echinochloa esculenta*) and foxtail millet (*Setaria italica*). The seeds were either purchased or collected in Japan. After filling the lower part of the pot with the seeds, water (ca. 900 ml), was added up to the inflection point below the rim. The pot was then placed on a fire made with broadleaf wood fuel until visible carbonised deposits had formed. The cooking continued for up to one hour. The temperature measured on the outer surface of the vessel often exceeded 300 °C. After the experiment the vessels were dried at room temperature. Then 1 g of ceramic was drilled from the ceramic interior at a depth between 2–5 mm.

### Lipid extraction

Lipids were extracted and methylated simultaneously using acidified methanol^49^. Briefly, methanol was added to homogenized charred deposits (1 mL to 10–30 mg) and ceramic powders drilled (d. 2–5 mm) from the sherd surface (4 mL to 1 g). The mixture was ultrasonicated for 15 min, and then acidified with concentrated sulphuric acid (200 μL). The acidified suspension was heated in sealed tubes for 4 h at 70 °C and then allowed to cool. Lipids were then extracted with *n*-hexane (3 × 2 mL) and dried under a gentle stream of N_2_. 10 μg of internal standard (*n*-hexatriacontane) was added to each sample prior to analysis by GC-MS and GC-C-IRMS using standard conditions and protocols^50^,^51^. In addition, the sherds from Majeon-ri were subjected to conventional solvent extraction following established methods^52^ to compare the recovery of miliacin between acidified methanol and solvent extraction. Sherd powders (1 g) were extracted by ultrasonication into a mixture of DCM:MeOH (2/1 v/v, 3 × 2 mL) to obtain the extracts which were combined and dried under a gentle stream of N_2_, followed by silylation with *N,O*-bis(trimethylsilyl)trifluoroacetamide (BSTFA) with 1% trimethylchlorosilane (TMCS) at 70 °C for one hour, and dried again under N_2_. *n*-hexane was added to the derivatised samples prior to gas chromatography-mass spectrometry (GC-MS).

### Gas Chromatography-Mass Spectrometry (GC-MS)

GC-MS was carried out on all samples using an Agilent 7890 A Series chromatograph attached to an Agilent 5975 C Inert XL mass-selective detector with a quadrupole mass analyser (Agilent technologies, Cheadle, Cheshire, UK). A splitless injector was used and maintained at 300 °C. The carrier gas used was helium, and inlet/column head-pressure was constant. The column (DB-5 ms) was coated with 5% phenyl-methylpolysiloxane column (30 m × 0.250 mm × 0.25 μm; J&W Scientific, Folsom, CA, USA). The oven temperature was set at 50 °C for 2 min, then raised by 10 °C min^−1^ until 325 °C was reached, where it was held for 15 min until the end of the run. The GC column was inserted directly into the ion source of the mass spectrometer. The ionisation energy of the mass spectrometer was 70 eV and spectra were obtained in scanning mode between *m/z* 50 and 800. The MS was also used in selected ion monitoring (SIM) mode with the oven temperature set at 50 °C for 1 min, then raised by 20 °C min^−1^ until 280 °C, then raised at 10 °C min^−1^ until reaching 325 °C, where it was held for 10 min until the end of the run. In SIM mode, a first group of ions (*m/z* 189, *m/z* 204, *m/z* 231, *m/z* 425, *m/z* 440) corresponding to miliacin fragmentation were monitored. After 16 min, a second group of ions (*m/z* 57, *m/z* 71, *m/z* 85, *m/z* 478, *m/z* 506) were monitored to record the internal standard.

### Gas Chromatography-combustion-Isotope Ratio Mass Spectrometry (GC-c-IRMS)

In each extract, compound specific stable carbon isotope analyses (GC-c-IRMS) of methyl palmitate (C_16:0_) and methyl stearate (C_18:0_) were conducted according to previous methodologies^53^. An Isoprime 100 (Isoprime, Cheadle, UK) linked to a Hewlett Packard 7890B series gas chromatograph (Agilent Technologies, Santa Clara, CA, USA) with an Isoprime GC5 interface (Isoprime, Cheadle, UK) was used. All samples were diluted with hexane and subsequently 1 μL of each sample was injected into a DB-5MS ultra-inert fused-silica column. The temperature was set for 0.5 min at 50 °C, and raised by 10 °C min-1 until 300 °C was reached, and held for 10 min. The carrier gas was ultra-high purity grade helium with a flow rate of 3 mL min^−1^. The gases eluting from the chromatographic column were split into two streams. One of these was directed into an Agilent 5975C inert mass spectrometer detector (MSD), for sample identification and quantification, while the other was directed through the GC5 furnace held at 850 °C to oxidise all carbon species to CO_2_. A clear resolution and baseline separation of the analysed peaks was achieved. Eluted products were ionized in the mass spectrometer by electron impact. Ion intensities of *m/z* 44, 45, and 46 were monitored in order to automatically compute the ^13^C/^12^C ratio of each peak in the extracts. Computations were made with IonVantage and IonOS Softwares (Isoprime, Cheadle, UK) and were based on comparisons with a standard reference gas (CO_2_) of known isotopic composition that was repeatedly measured. The results from the analysis are reported in parts per mil (‰) relative to an international standard (V-PDB). Replicate measurements of each sample and a mixture of fatty acid methyl esters (FAMEs) with δ^13^C values traceable to international standards were used to determine instrument precision (±0.3‰) and accuracy (±0.5‰) standard deviation. Finally a standard mixture of C_16:0_ and C_18:0_ fatty acids of known isotopic composition processed in each batch under identical conditions was used to correct for the methylation of the carboxyl group that occurs during acid extraction.

### Elemental Analysis Isotope Ratio Mass Spectrometry

About 1 mg of crushed and homogenised charred deposit was weighed into tin capsules in duplicate. These were directly analysed by elemental analysis - isotope ratio mass spectrometry (EA-IRMS) to determine their bulk stable carbon (δ^13^C) and nitrogen isotope (δ^15^N) composition, as previously reported^46^. Samples yielding less than 1% N were not used and instrument precision on repeated measurements was ±0.2‰ (s.e.m.). δ^13^C, δ^15^N = [(R_sample_/R_standard_−1)] × 1,000, where R = ^13^C/^12^C and ^15^N/^14^N. All sample measurements are expressed in per mil relative to the δ^13^C standard (Vienna PeeDee Belemnite, V-PDB) and the δ^15^N standard (air N_2_) respectively.

## Additional Information

**How to cite this article**: Heron, C. *et al*. First molecular and isotopic evidence of millet processing in prehistoric pottery vessels. *Sci. Rep.* **6**, 38767; doi: 10.1038/srep38767 (2016).

**Publisher's note:** Springer Nature remains neutral with regard to jurisdictional claims in published maps and institutional affiliations.

## Supplementary Material

Supplementary Information

## Figures and Tables

**Table 1:** Summary of bulk isotope data from Early Bronze Age (EBA), mostly EBA and Late Bronze Age/Early Iron Age (LBA/EIA) samples from Bruszczewo.

	*EBA* (*n* = *24*)		*‘Mostly EBA’* (*n* = *15*)		*LBA/EIA* (*n* = *22*)	
	*δ*^*13*^*C* (‰)	*δ*^*15*^*N* (‰)	*δ*^*13*^*C* (‰)	*δ*^*15*^*N* (‰)	*δ*^*13*^*C* (‰)	*δ*^*15*^*N* (‰)
Mean (1 s.d.)	−25.3 ± 1.0	5.9 ± 1.1	−23.9 ± 3.4	4.4 ± 1.9	−20.8 ± 4.2	4.8 ± 2.5
Range	−24.0 to −27.8	3.8 to 8.5	−13.1 to −26.6	1.0 to 8.4	−11.8 to −27.2	1.1 to 12.6
Atomic C:N ratio	17.8 ± 9.1		20.4 ± 10.3		19.2 ± 7.6	

**Table 2:** Bulk isotope data obtained on one EBA and one LBA/EIA sample selected for GC-MS and GC-C-IRMS.

	C (%)	N (%)	δ^13^C (‰)	δ^15^N (‰)	C/N ratio
EBA (F1380ID1776)	39.8	4.2	−26.0	5.8	11.1
LBA/EIA (F5017ID4992)	40.1	2.2	−11.8	4.6	21.3

**Table 3:** Compositional data, bulk and compound-specific δ^13^C values for one probable C_3_ and one probable C_4_ sample from Bruszczewo.

	Compositional data (GC-MS)	*δ*^*13*^*C*_*BULK*_ (‰)	*δ*^*13*^*C*_*16:0*_ (‰)	*δ*^*13*^*C*_*18:0*_ (‰)	*Δ*_*BULK*_*-*_*CSIA*_	Data from Ballentine *et al*.^46^
EBA (F1380ID1776) Probable C_3_ plant residue	Miliacin absent; ω-(o-alkylphenyl)alkanoic acids (C18 present, C16 trace only); C_14:0_ (10.2%), C_16:0_ (75.1%), C_18:0_ (14.7%)	−26.0	−30.3	−31.2	4.7	3–9‰ depletion in δ^13^C values of fatty acids relative to the bulk C_3_ plant
LBA/EIA (F5017ID4992) Probable C_4_ plant residue	Miliacin present; ω-(o-alkylphenyl)alkanoic acids (C18 present, C16 trace only); C_14:0_ (5.3%), C_16:0_ (72.9%), C_18:0_ (21.8%)	−11.8	−23.7	−23.7	11.9	7–14‰ depletion in δ^13^C values of fatty acids relative to the bulk C_4_ plant

Both samples also yielded low levels of *n*-alkenoic acids, odd-carbon number *n*-alkanoic acids and dicarboxylic acids. The Δ_BULK_-_CSIA_ value expresses the difference between the bulk carbon isotope value and the mean of the compound-specific carbon isotope values obtained on the C_16:0_ and C_18:0_ *n*-alkanoic acids. These data are compared with depletions in *n*-alkanoic acids extracted from authentic C_3_ and C_4_ plants relative to bulk tissue values using literature data^46^. Compound-specific carbon isotope data have been reported for miliacin extracted from lake sediments and compared with authentic samples of *P. miliaceum*^54^.
