# Supplementary material for: First molecular and isotopic evidence of millet processing in prehistoric pottery vessels
Source: Sci Rep. 2016 Dec 22;6:38767. doi: 10.1038/srep38767 (PMC5177950; doi:10.1038/srep38767)
Supplement: Supplementary Information [file srep38767-s1.pdf]

Supplementary Information

First molecular and isotopic evidence of millet processing in prehistoric pottery vessels

Carl Heron, Shinya Shoda, Adrià Breu Barcons, Janusz Czebreszuk, Yvette Eley, Marise Gorton, Wiebke Kirleis, Jutta Kneisel, Alexandre Lucquin, Johannes Müller, Yastami Nishida Joon-ho Son, Oliver E. Craig

Supplementary Table 1 – Heron *et al.*
First molecular and isotopic evidence of millet processing in prehistoric pottery vessels

		$\delta^{13}C$ (‰)	% Carbon	$\delta^{15}N$ (‰)	% Nitrogen	Atomic C/N ratio
**EBA (*n* = 24)**						
	F14076ID12960	-26.2	16.9	5	2.2	8.8
	F6122ID7932	-27.8	34.3	6.4	3.7	11
	F4311ID4409	-24	45.8	5.2	4.6	11.6
	F14200ID14074	-24.8	29.1	6.1	1	34.7
	F11808ID12284	-24.2	20.1	5.3	1.3	18.7
	F11806ID12385	-26.4	27.2	4.8	1.6	20.6
	F14076ID12960	-26.2	24.3	5.0	2.2	12.5
	F11199ID9973	-26.7	42.5	4	1.1	44.3
	F6152ID7884	-25.3	51.5	5.6	2.9	20.4
	F8276ID6467	-24.6	55.4	3.8	2.1	30.8
	F8506ID6567	-24.8	30.6	6.7	3	12.1
	F8513ID6694	-25.0	39.8	7	7.8	5.9
	F8546ID6561	-25.0	47	6.1	3.4	16.3
	F8556ID6708	-26.4	41	6.8	3.3	14.3
	F8595ID6697	-24.3	47.2	4.9	2.3	24.6
	F1138ID2847	-25.2	33.8	7	3.2	12.3
	F1380ID1776	-26.0	39.8	5.8	4.2	11.1
	F14646ID14639	-24.7	32.5	6.5	2.3	16.9
	F14697ID14664	-24.4	32.3	8.5	3.4	11.2
	F2149ID1267	-24.3	45.7	6.2	3.2	16.6
	F4308ID4473	-24.9	45.6	6.4	2.5	21.1
	F4308ID4475	-26.2	35.8	6.8	5.4	7.6
	F4420ID4357	-25.2	35	4.2	1.4	29.2
	F4424ID4350	-24.5	44	7.1	3.3	15.3
	Mean	-25.3	37.4	5.9	3	17.8
	Standard deviation	1.0	9.8	1.1	1.5	9.1

**Mostly EBA (*n* = 15)**						
	F4401ID4376	-24.4	54	6.8	4.1	15.5
	F11154ID10227	-23.6	34.5	8.4	5.4	7.3
	F7164ID2984	-26.6	33.6	3.9	1.2	31.1
	F9105ID7293	-13.1	47.8	3.9	2.8	19.9
	F1103ID2782	-24.3	34.7	5.4	3.6	11.1
	F4141ID3941	-22.3	28	5.4	3.1	10.6
	F4203ID4093	-25.2	22.1	2.6	1.8	14.2
	F4207ID4257	-24.6	43.6	4.9	2.1	24.2
	F4354ID4388	-24.5	39.5	2.4	1.6	29.9
	F4399ID4564	-24.8	44.1	5.8	2.6	19.4
	F4418ID4429	-24.6	47.9	3.5	3.2	13.8
	F7173ID2933	-26.6	33.6	3.9	1.2	31.1
	F2336ID1460	-26.6	16.4	4.3	1.3	15.2
	F7170ID4890	-26.5	38.7	1	1	46.1
	F3114ID521	-20.3	35.8	4.8	2.5	16.6
	Mean	-23.9	37	4.5	2.5	20.4
	Standard deviation	3.4	10	1.8	1.2	10.3
**LBA/EIA (*n* = 22)**						
	F5044ID5396	-18.3	48.1	3.8	3	19
	F5018ID5071	-27.2	34.6	0.7	1.4	19.2
	F4382ID4528	-23.9	34.4	7	4	9.9
	F4096ID3693	-25.5	50.1	5.8	5.1	11.6
	F4088ID3699	-16.6	49.3	4.8	1.6	37.4
	F2344ID1523	-25.8	31.8	5.1	2.3	16.6
	F1428ID1810	-18.9	44.7	2.9	2.3	23.3
	F12384ID18300	-26.5	23.9	4.7	1	28.4
	F1045bID2594	-18.6	47.3	7.6	4.2	13.1
	F5017ID4992	-11.8	23.9	4.6	2.2	20.9

	F5020ID5135	-15.5	43.9	3.3	3.1	16.6
	F6057ID7828	-20.4	38.7	3.8	2.2	20.2
	F7061ID4765	-23.4	38.1	12.6	5.1	8.8
	F7063ID4698	-15.3	53.9	2.8	2.3	28.1
	F9171ID7454	-22.8	42.9	6.4	2.2	22.2
	F1032ID2277	-16	48.2	3.9	2.7	21.2
	F1033ID1943	-21.5	46.9	3.5	5.1	10.9
	F4088ID3707	-19.3	19.4	8.1	1.4	19.4
	F4155ID3875	-19.4	42.1	1.1	4	12.1
	F4187ID4127	-20.5	36.5	4.6	5.1	8.4
	F4211ID4280	-24.8	36.7	4.5	1.6	27.8
	F4378ID4377	-24.8	53.6	3	2.2	27.9
	Mean	-20.8	40.4	4.8	2.9	19.2
	Standard deviation	4.2	9.6	2.5	1.3	7.6

Supplementary Table 1 - Bulk carbon and nitrogen isotope data obtained on sixty-one charred visible deposits (foodcrusts) samples Bruszczewo, Poland

Lab. Code	Site	Period	Lipid conc. (μg g^{-1})	Major compounds detected	miliacin (AME)	miliacin (TLE)	$C_{16:0}$ $\delta^{13}C$ (‰)	$C_{18:0}$ $\delta^{13}C$ (‰)
MJR01	Majeon-ri	LBA	35	FA($C_{10:0-26:0}$, $C_{16:1-18:1}$), DC(C_{10}), cholesterol, β-sitosterol, *n*-alkanol (C_{14-30})			-29.0	-29.4
MJR02	Majeon-ri	LBA	120	FA($C_{9:0-28:0}$, $C_{18:1}$, C_{17br}), DC($C_{6,7,14}$), DAG, *n* -alkanol (C_{24-36}), K_{31}	Y		-27.3	-25.6
MJR03	Majeon-ri	LBA	29	FA($C_{11:0-30:0}$, $C_{18:1-22:1}$, $C_{12,15,17br}$), DC($C_{8,9}$), levoglucosan cholesterol, β-sitosterol,, 1-alkanol (C_{14-30})	Y	Y		
MJR04	Majeon-ri	LBA	109	FA($C_{10:0-26:0}$, $C_{18:1}$, C_{17br}), DC(C_{9-12}), phy, cholesterol, campesterol, β-sitosterol			-26.8	-25.7
MJR05	Majeon-ri	LBA	20	FA($C_{12:0-24:0}$, $C_{18:1-22:1}$, $C_{15,17br}$), cholesterol, campesterol, β-sitosterol				
MJR06	Majeon-ri	LBA	155	FA($C_{10:0-30:0}$, $C_{18:1-22:1}$, C_{15br}), DC(C_{7-13}), APFA(C_{18}), phy, β-sitosterol(tr), stigmastanol			-19.6	-17.9
MJR07	Majeon-ri	LBA	25	FA($C_{12:0-26:0}$, $C_{16:1-22:1}$, $C_{15,17br}$), DC(C_{9}), cholesterol (tr)				
MJR08	Majeon-ri	LBA	38	FA($C_{12:0-30:0}$, $C_{18:1-22:1}$, $C_{15,17br}$), DC(C_{9}), APFA(C_{18tr}), cholesterol, cholestanol, campesterol, stigmasterol, β-sitosterol, stigmastanol,	Y	Y	-26.8	-27.1
MJR09	Majeon-ri	LBA	117	FA($C_{14:0-30:0}$, $C_{18:1-22:1}$, C_{17br}), DC(C_{9-11}), *n*-alkane (C_{25-33}), *n*-alkanol (C_{24-36}), stigmastanol, cholestanone, stigmastanone, K_{31}	Y	Y	-24.9	-21.6
MJR10	Majeon-ri	LBA	123	FA($C_{8:0-30:0}$, $C_{18:1}$, C_{17br}), DC(C_{9}), stigmastanol	Y	Y	-19.3	-20.0
MJR11	Majeon-ri	LBA	19	FA($C_{14:0-26:0}$, $C_{18:1-22:1}$, $C_{15,17br}$)	Y			
MJR12	Majeon-ri	LBA	32	FA($C_{14:0-26:0}$, $C_{18:1-22:1}$, $C_{15,17br}$), DC(C_{9}), cholesterol	Y	Y		
MJR13	Majeon-ri	LBA	17	FA($C_{14:0-18:0}$, $C_{18:1-22:1}$, C_{17br}), cholesterol				
MJR14	Majeon-ri	LBA	19	FA($C_{14:0-24:0}$, $C_{16:1-22:1}$, $C_{15,17br}$), cholesterol				
MJR15	Majeon-ri	LBA	23	FA($C_{14:0-26:0}$, $C_{18:1-22:1}$, C_{15br}), cholesterol, β-sitosterol (tr)				

Supplementary Table 2 – Molecular and compound-specific carbon isotope data obtained by analysis of pottery vessel sherds from Majeon-ri, Korea. Key to the molecular data: FA($C_{n:x}$) – fatty carboxylic acids with carbon length *n* and number of unsaturations *x*, branched-chain carboxylic acids identified as FA($C_{n\mathrm{br}}$); *n*-alkanol – alkanol with *n* carbon atoms, DC - α,ω-dicarboxylic acids of chain length C_n; APFA - ω-(*o*-alkylphenyl)alkanoic acids of chain length C_n; DAG – diacylglycerol; K_{31} – mid-chain ketone with chain-length of 31 carbon atoms; phy – phytanic acid. Detection of miliacin is shown according to extraction method, either acid-methanol (AME) and solvent extraction (TLE).
